# Refractory IgD Multiple Myeloma Treated with Daratumumab: A Case Report and Literature Review

**DOI:** 10.1155/2016/2490168

**Published:** 2016-09-26

**Authors:** Muhammad Husnain, Sandra Kurtin, Nikki Barkett, Irbaz Bin Riaz, Amit Agarwal

**Affiliations:** ^1^Department of Medicine, University of Arizona, Tucson, AZ, USA; ^2^Banner University Medical Center Tucson, Department of Hematology Oncology, University of Arizona, Tucson, AZ, USA

## Abstract

Patients with relapsed and refractory multiple myeloma have poor prognosis. A recent analysis of patients with relapsed and refractory multiple myeloma who were refractory to both proteasome inhibitors and immunomodulatory drugs showed the median overall survival of 9 months only. Daratumumab is the first-in-class human monoclonal antibody against CD38 cells which was studied in phase I/II trials for treatment of these patients with relapsed refractory multiple myeloma. It showed an overall response rate of 36% and a median overall survival (OS) of 17 months in these patients. We report a case of 40-year-old man with immunoglobulin D (IgD) multiple myeloma whose disease was refractory to at least 5 different chemotherapy regimens including proteasome inhibitors and immunomodulatory drugs. The clinical studies assessing daratumumab did not include any patients with IgD myeloma which is a rare form of multiple myeloma and to our knowledge is the first study reporting use of daratumumab in IgD myeloma.

## 1. Introduction

The conventional treatment options in relapsed multiple myeloma include hematopoietic cell transplantation (HCT) or trial of the previously tried chemotherapy regimens. Several new drugs such as Panobinostat (first-in-class histone deacetylase inhibitor), daratumumab (the first monoclonal antibody), ixazomib (the first oral proteasome inhibitor), and Elotuzumab (the first-in-class immunostimulatory agent) have been approved in the past year [1–4]. However, there is limited experience with the use of these novel drugs in the real life clinical setting and there are no published reports of real life experience with these drugs since their approval. Daratumumab has shown promising results in clinical trials in the setting of relapsed refractory multiple myeloma but most of the patients in clinical trials were IgG, IgA, or Bence Jones proteins multiple myeloma. There is little data regarding daratumumab role in the setting of IgD multiple myeloma. We present a case of Immunoglobulin D (IgD) multiple myeloma which was refractory to at least five different regimens and finally responded when treated with daratumumab.

## 2. Case Presentation

A 40-year-old man with a known diagnosis of Immunoglobulin D (IgD) lambda multiple myeloma presented with relapsed multiple myeloma (MM). He first presented in September 2007 with the chief complaint of cough and left sided abdominal pain. Physical examination was significant for splenomegaly. His labs were significant for pancytopenia. A bone marrow biopsy in September 2007 was significant for 100% cellularity and sheets of atypical cells positive for CD138 and lambda light chains consistent with multiple myeloma. Protein electrophoresis showed a monoclonal spike (M spike) and an elevated IgD level of 190 mg/L. A diagnosis of IgD multiple myeloma was made and he was initially started on dexamethasone and later in October 2007 thalidomide was added to his regimen. Patient had intermittent lapses in his thalidomide treatment because of his insurance issues and his IgD level slowly kept rising. In July 2008 his IgD level was found to be 293 mg/L. A bone marrow biopsy performed in July 2008 was significant for 5% plasma cells by immunohistochemistry. He was continued on dexamethasone and thalidomide. A repeat bone marrow biopsy in July 2009 showed 40% plasma cells. At that point bortezomib was added to his regimen to reduce his tumor burden and he was referred for autologous peripheral blood stem cell transplantation (PBSCT). Patient only took 50 mg of thalidomide instead of usual 100 mg. His repeat bone marrow biopsy in February 2010 showed persistent multiple myeloma with 20% monoclonal plasma cells with overall cellularity of 60%. In March 2010 his IgD level increased up to 531 mg/L. At that point his thalidomide dose was increased to 200 mg daily. Patient was having issues with compliance. Eventually, he underwent autologous peripheral blood stem cell transplant (PBSCT) on January 2011 after 6 cycles of salvage CVAD (cyclophosphamide, vincristine, doxorubicin, and dexamethasone) chemotherapy. He was transplanted with stable disease with his pretransplant IgD level of 112 mg/L. He did not receive maintenance therapy and only 10 months after transplant there was evidence of myeloma progression with IgD level increasing to over 500 mg/L in October 2011. Patient at that point chose to pursue alternative therapies and was lost to follow-up. He presented again in April 2012 with severe lower back pain and an IgD level of 4020 mg/L. He was immediately started on pulse dose dexamethasone and lenalidomide was added in May 2012 (25 mg daily for 21 of 28 days). His IgD level decreased to 60 mg/L in November 2012 consistent with a very good partial response (VGPR) and he underwent a second autologous PBSCT in January 2013. His posttransplant course was complicated by some chemotherapy associated nausea, diarrhea, and neutropenic fever. He recovered well and was discharged home in stable condition. His day 100 bone marrow biopsy in April 2013 showed 20–40% plasma cells and an IgD level of 107 mg/L consistent with disease progression. Patient was restarted on lenalidomide 25 mg daily and had an initial response with a decrease in his IgD, which nadired at about 23.9 mg/L in June 2013 but his IgD quickly began to rise again.

**Figure 1:**
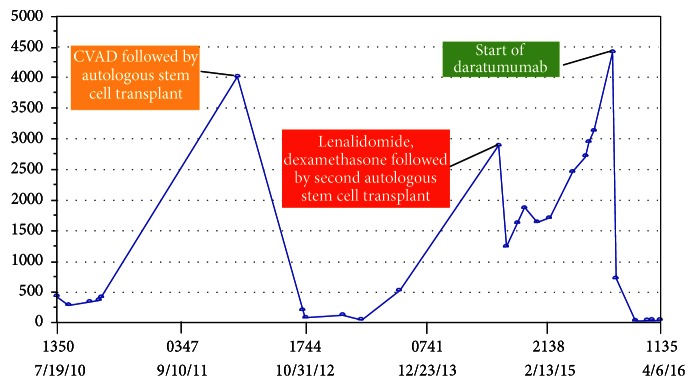
IgD levels over the course of 6 years of treatment.

**Figure 2:**
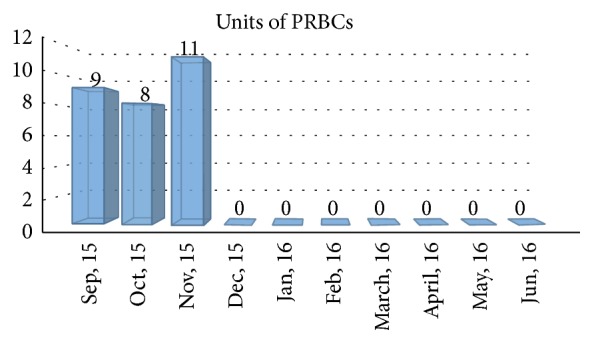
Transfusion requirements prior to starting daratumumab and after daratumumab.

Despite lenalidomide and dexamethasone, his IgD continued to rise to as high as 500 mg/L and at that point he was started on Pomalidomide in December 2013. He completed 2 cycles of Pomalidomide and dexamethasone but his disease continued to progress so he was switched to bortezomib, Pomalidomide, and dexamethasone in September 2014 for about 4-5 months and later carfilzomib, Pomalidomide, and dexamethasone for an additional 5 months from January 2015 to May 2015 without much improvement. He was started on bortezomib, Panobinostat, and dexamethasone in July 2015 but could not tolerate this regimen. He was deemed to be unsuitable for allogeneic stem cell transplant by his insurance. He was requiring PRBC transfusion twice a week to maintain his hemoglobin to >7. He was not a candidate for clinical trials because of his cytopenias. At that point we decided to treat him with daratumumab. His pretreatment IgD levels in December 2015 were 4000 mg/L and came down drastically to 5 mg/L in May 2016 after 5 cycles of daratumumab showing very good partial response (Figure 1). During his first infusion, he developed a grade 3 infusion reaction. His other side effects to medication were transient neutropenia and* E. coli* and* Giardia* diarrhea for which he was treated with antibiotics. His transfusion requirements which had been very high throughout his disease course requiring multiple transfusions with packed red blood cells (PRBCs) also came down dramatically with stable hemoglobin levels after the start of daratumumab (Figure 2). Patient continues on daratumumab, with his last IgD level at 12 mg/L in June 2016.

## 3. Discussion

Multiple myeloma is a plasma cell neoplasm with an incidence of 6.3 cases per 100,000 persons per year in United States [5, 6]. The treatment outcome and overall survival have significantly improved after the use of novel agents and autologous stem cell transplant for multiple myeloma [1]. However, for patients who are refractory to these agents, the prognosis remains poor with overall survival (OS) of 9 months only [7]. This clearly demonstrates the need for additional agents with novel mechanisms of actions. In the past decade, many new potential therapeutic targets have been identified, including histone deacetylation, proteasome activity, mammalian target of rapamycin, and targetable surface receptors such as SLAMF-7, CD38, and CD40 [1, 2, 8]. Monoclonal antibodies in particular present a new exciting approach. Two new monoclonal antibodies daratumumab (anti-CD38) and Elotuzumab (anti-SLAMF-7) have been approved by FDA for relapsed refractory multiple myeloma [9].

CD38 is a transmembrane glycoprotein that is highly expressed on multiple myeloma cells and at low levels on normal lymphoid and myeloid cells [10, 11]. It functions as an ectoenzyme involved in regulating intracytoplasmic concentration of calcium and the catabolism of extracellular nucleotides. Daratumumab, an IgG1-k fully human anti-CD38 monoclonal antibody, exerts its cytotoxic effect through a number of mechanisms after binding to CD38, including antibody-dependent cell-mediated cytotoxicity (ADCC), complement-dependent cytotoxicity (CDC), antibody-dependent cell-mediated phagocytosis (ADCP), and the direct induction of apoptosis [12, 13]. Moreover, an immunomodulatory effect of daratumumab was also recently described [14]. The safety of daratumumab was investigated in a dose-escalation phase I/II trial of 104 patients with relapsed or refractory multiple myeloma. The most common adverse events were grade 3 or 4 pneumonia and thrombocytopenia. The overall response rate was 36% in the cohort that received 16 mg per kilogram and 10% in the cohort that received 8 mg per kilogram. In the cohort that received 16 mg per kilogram, the median progression-free survival was 5.6 months [11]. In another open label phase II trial (SIRIUS) 106 patients with multiple myeloma who had failed at least three lines of treatment including a proteasome inhibitor and immunomodulatory drugs were treated with daratumumab [15]. Two dose levels 8 mg/kg and 16 mg/kg were evaluated. Daratumumab was well tolerated and there were no discontinuations due to drug-related treatment emergent adverse effects. Infusion reactions were seen in 45 (42%) patients but these were mostly grades 1 and 2 with five patients reporting grade 3 and none with grade 4 infusion reaction. Other side effects included fatigue (40%) and anemia (33%). Patients treated with daratumumab showed 29% overall response with a median time to first response of one month. The estimated median progression-free and overall survival rates were 3.7 and 17.5 months, respectively [15].

Two phase III trials [16] are evaluating patients with relapsed or refractory disease; one is comparing daratumumab plus bortezomib and dexamethasone with bortezomib and dexamethasone (NCT02136134) [16], and the other is comparing daratumumab plus lenalidomide and dexamethasone with lenalidomide and dexamethasone (NCT02076009) [16]. The final results of these studies are awaited but the CASTOR trial (Velcade/dex versus Dara/Velcade/dex) was stopped early because at an interim analysis the study met its end point with a very impressive hazard ratio of 0.39 favoring the daratumumab arm. The POLLUX study (Rev/dex versus Dara/Rev/dex) has also shown similarly impressive results with a hazard ratio of 0.37 favoring the Dara arm. Two additional phase III studies are being performed in patients with previously untreated multiple myeloma; one is comparing daratumumab plus bortezomib, melphalan, and prednisone with bortezomib, melphalan, and prednisone (NCT02195479), and the other is evaluating daratumumab plus lenalidomide and dexamethasone with lenalidomide and dexamethasone (NCT02252172) [17].

## 4. Conclusions

Despite significant progress in the treatment of multiple myeloma, the disease remains incurable in a vast majority of patients. The approval of promising new agents will undoubtedly improve outcomes for myeloma patients. Daratumumab is a monoclonal antibody that is now approved for treatment of multiple myeloma patients and has shown significant response in the real world setting. Our case illustrates the potential of a dramatic response using monoclonal antibodies in patients that are heavily pretreated and having a very meaningful impact towards their outcome.
